# Three reasons protein disorder analysis makes more sense in the light of collagen

**DOI:** 10.1002/pro.2913

**Published:** 2016-04-19

**Authors:** Ben Smithers, Matt E. Oates, Peter Tompa, Julian Gough

**Affiliations:** ^1^ Department of Computer ScienceUniversity of BristolBristolBS8 1UBUnited Kingdom; ^2^ VIB Structural Biology Research Center (SBRC)Vrije Universiteit BrusselBrussels1050Belgium

**Keywords:** intrinsically disordered proteins, collagen helix, exons, phase symmetry, splicing

## Abstract

We have identified that the collagen helix has the potential to be disruptive to analyses of intrinsically disordered proteins. The collagen helix is an extended fibrous structure that is both promiscuous and repetitive. Whilst its sequence is predicted to be disordered, this type of protein structure is not typically considered as intrinsic disorder. Here, we show that collagen‐encoding proteins skew the distribution of exon lengths in genes. We find that previous results, demonstrating that exons encoding disordered regions are more likely to be symmetric, are due to the abundance of the collagen helix. Other related results, showing increased levels of alternative splicing in disorder‐encoding exons, still hold after considering collagen‐containing proteins. Aside from analyses of exons, we find that the set of proteins that contain collagen significantly alters the amino acid composition of regions predicted as disordered. We conclude that research in this area should be conducted in the light of the collagen helix.

## Introduction

Intrinsically disordered proteins, or regions of proteins, are those that lack a stable three‐dimensional structure under natural conditions. A prediction of intrinsic disorder now accounts for around a third of the eukaryotic proteome,^1^, ^2^ whilst disorder is highly important for a variety of functions including cell signalling and regulation.^3^, ^4^ Intrinsically disordered protein sequence is compositionally biased and characterized by the increased occurrence of charged and polar residues known as disorder‐promoting amino acids.^5^, ^6^

The intron–exon architecture of eukaryotic genes increases the diversity of the proteome through alternative splicing, whereby exons are selectively included to create multiple protein products from the same gene.^7^, ^8^ Exons contributing to all proteins transcribed from a given gene are termed constitutive. Additionally, exons may be classified as symmetric if their flanking introns have the same phase. The phase of an intron is given by its position relative to the codons of the coding sequence: an intron falling between two codons is phase 0, an intron occurring after one base of a codon is phase 1; an intron occurring after two bases of a codon is phase 2. Thus a symmetric exon contains a multiple of three nucleotides and the duplication of such exons does not disrupt the reading frame of a sequence.

Recently, a number of links between protein disorder and exons have been established. For example, alternatively spliced exons are enriched for protein disorder, which is thought to be key for tissue‐specific protein interactions and cell signalling.^9^, ^10^ The reduced structural constraints of intrinsically disordered regions may make them more suited to the conditional presence of alternatively spliced sequence, or indeed exons read in multiple reading frames.^11^, ^12^ Furthermore, the locations of splice junctions themselves may be constrained by the tolerance for disorder‐promoting amino acids in the translated protein product.^13^ Finally, it was recently shown that symmetric exons are also enriched for protein disorder, with the suggestion that this symmetry has aided the modular evolution of the human proteome.^14^ This previous work includes a graph of the distribution of the lengths of exons that encode disordered protein regions (Fig. 3 of Ref. 14), which contains a striking feature: peaks in the distribution suggest certain lengths of exons are much more common, particularly exons of 18 amino acids in length. Indeed, this peak is identifiable in the length distribution of all exons—that is not just those encoding protein disorder—and has been observed in the literature at least as far back as 1995, without apparent explanation.^15^ Similar graphs are independently reproduced here for exons encoding protein disorder (Fig. 2) and for all exons (Supporting Information Fig. S1) in the human genome.

We have extended the original work and now identified that this peak, long known in the literature, can be attributed to exons that encode the collagen triple helix. The collagen triple helix is an extended, fibrous structure created from three α chains. Each chain forms a left‐handed helix, which wrap around each other to form a larger, right‐handed helix.^16^, ^17^, ^18^, ^19^ As a major component of the extracellular matrix, collagen is the most abundant protein in mammals.^17^, ^19^ However, the collagen triple‐helix structure is also found in a variety of non‐collagenous proteins.^18^, ^19^ Collagen‐like sequence is found in 0.4% of human genes and proteins, with these proteins accounting for 0.9% of all amino acids in the proteome. A key signature of collagen α chains is the repetitive motif G‐X‐Y, where glycine is found at every 3rd position with X and Y as any amino acid but frequently proline and hydroxyproline respectively.^17^, ^18^

Perhaps as a result of this highly biased composition and structure‐breaking nature of both glycine and proline, collagen proteins have previously been found to have high levels of predicted intrinsic disorder.^20^ However, fibrous proteins such as collagen are typically not considered to be the same type of structure as intrinsic disorder. The latest version of the structural classification of proteins (SCOP2) recognizes four types of structure: globular, transmembrane, fibrous and intrinsically disordered, a viewpoint shared by others in the field.^21^, ^22^ However, the individual monomeric chains may be considered intrinsically disordered prior to the formation of the triple‐helix structure.^23^

It is known that collagen genes contain a large number of exons predominantly of length 54 nucleotides (18 amino acids), with 36 and 45 nucleotide exons also being common.^17^, ^24^ However the link between collagen and the unusual peaks in the distribution of exon lengths has not previously been identified. In addition to this, the collagen helix has been noted to contribute to a cyclic pattern of glycine usage at the start and end of exons.^25^ As such, we wished to consider what other published results may be affected by the collagen helix.

Firstly we determine how frequently the collagen helix is found within the human genome and to what extent it is predicted to be disordered. We then demonstrate the impact this structure has on the distribution of exon lengths, before considering if the enrichment for protein disorder amongst symmetric and alternative exons may be related to collagen‐encoding exons. Finally, we look at the amino acid distribution of disordered proteins to investigate potential effects of the collagen helix that are unrelated to exons or splicing.

## Results

### The collagen helix is found widely across the human genome, in genes with many exons and is predicted disordered

We found significant assignments (*E*‐value < 0.0001) to the collagen helix Pfam (PF01391^26^) in 361 human proteins transcribed from 89 genes, equivalent to 0.4% of the total of each within the genome.

Each collagen‐containing protein is typically encoded on many exons—28.8 exons per protein, compared to 8.2 exons for other proteins in the human genome (*P* = ∼0). Throughout the rest of this work, we consider the impact of the collagen helix by comparison to a set excluding collagen. We define a protein as collagen‐containing if it has at least one copy of the collagen Pfam domain, and exclude all exons from that protein from the collagen‐free set. This corresponds to 10,389 exons, which is 1.4% of the total number of exons in all transcripts in the genome.

**Figure 1:**
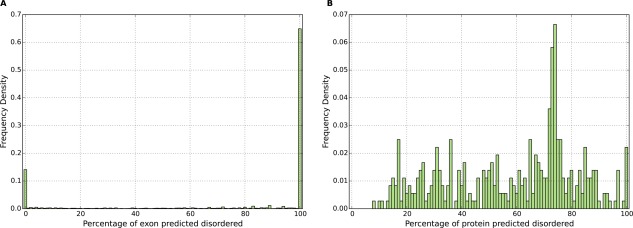
Distribution of the percentage disorder of proteins encoding at least one collagen helix (Pfam domain PF01391) in the human genome. In figure A on the left, the disorder percentage of exons encoding these proteins is shown; in figure B on the right, the disorder percentage of each protein is shown.

Exons in collagens typically encode either fully ordered (14.1%) or fully disordered (64.9%) protein, as shown in Figure 1(A). However in line with previous literature, we classify exons as mostly ordered and mostly disordered when <30%, or >70%, of residues are predicted disordered.^14^ These thresholds correspond to 19.9 and 73.7% of exons in collagen‐containing proteins, respectively. Thus there are 7,652 mostly disordered exons within collagen‐containing proteins, which represents 7.9% of all mostly disordered exons in the human proteome.

Considering whole proteins, there is more spread in the percentage of residues predicted disordered as shown in Figure 1(B)—an average of 58.3% of residues are predicted disordered among all collagen proteins. We note that disorder levels are likely to be somewhat conservative, owing to the use of a consensus of multiple predictors available in the D^2^P^2^ database.^2^

### Exons encoding the collagen helix are responsible for the peaks in exon length distribution

**Figure 2:**
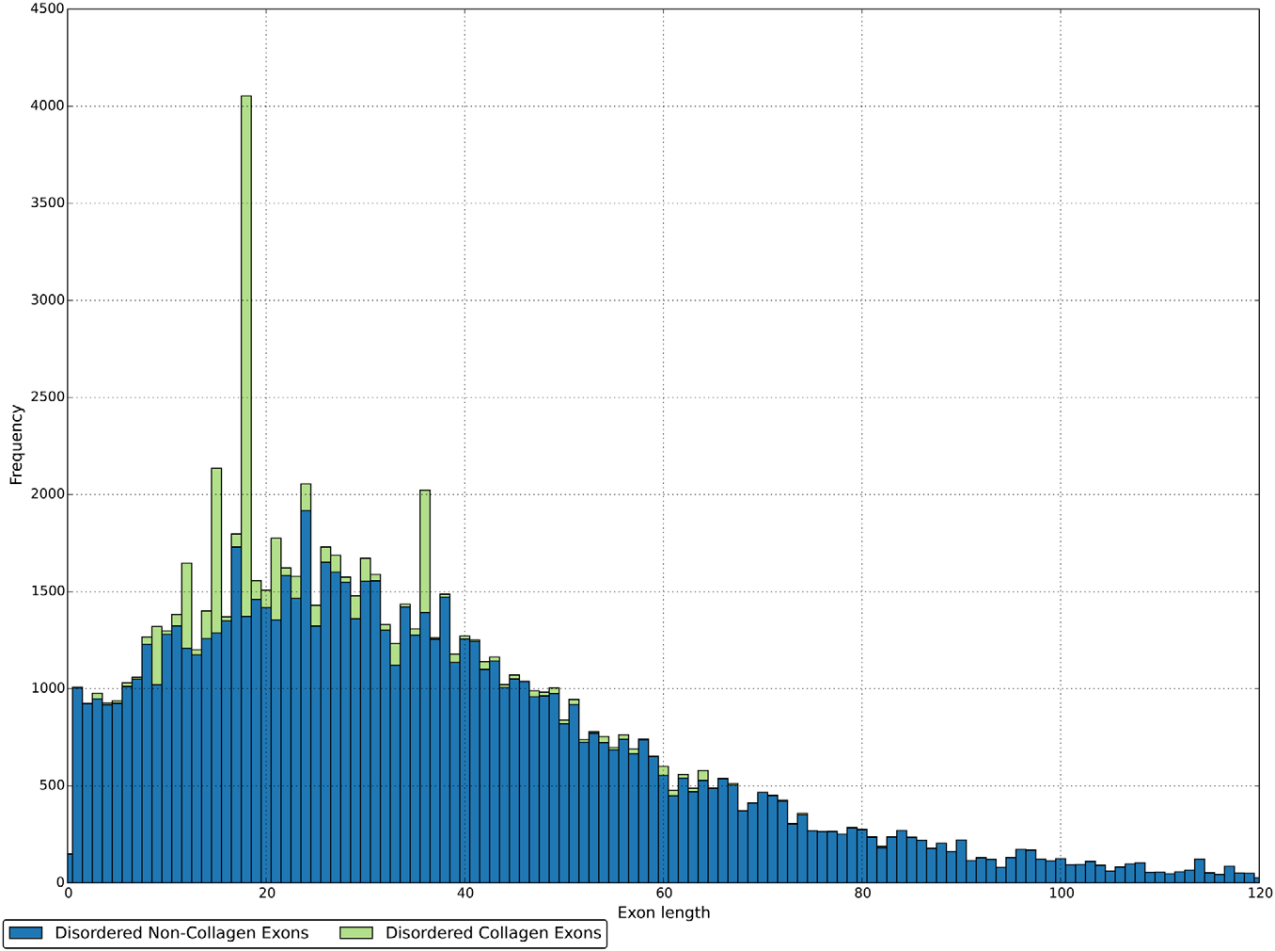
The length distribution of disorder encoding exons. Exons that are part of a collagen‐containing protein are shown in green; exons not in collagen‐containing proteins are show in blue. The distributions are stacked such that the length distribution of all disorder encoding exons is given by the height of both distributions.

We compared the distribution of exon lengths for all exons in the human genome with all exons excluding those encoding collagen proteins. Figure 2 shows that collagen encoding proteins account for the major peaks in the length distribution of mostly disordered exons, in particular at 12, 15, 18, and 36 residues. Of the 7,652 mostly disordered exons that encode collagen proteins, 4,595 are of these four specific lengths.

Removing collagen‐encoding proteins significantly alters the distribution of exon lengths (Kolmogorov–Smirnov test, *P* < 1.78E‐43). In addition, there is a small but significant increase in the mean exon length, from 51.1 to 53.5 amino acids (*P* < 3.27E‐6).

After excluding collagen‐encoding proteins, visual inspection suggests that two peaks remain in the distribution of exon lengths: at 17 and 24 residues. After clustering the amino acid sequences encoded by exons of these sizes, we found that the largest clusters in both cases are exons found in proteins containing Pfam domain PF06758, a repeat of unknown function. Exons of these sizes are typically found between occurrences of the domain. Nonetheless, after excluding proteins encoding this domain, both peaks remain evident (Supporting Information Fig. S2). Thus neither appears wholly attributable to a structural feature in the same manner as the peaks associated with the collagen helix.

### Symmetric exons are not enriched for protein disorder, after excluding collagen proteins

It was previously reported that higher levels of phase symmetry are found in exons that encode mostly disordered protein regions.^14^ We have found that this effect is due to collagen‐encoding proteins, as summarized in Table 1. Within our dataset, 46.27% of mostly disordered exons are symmetric compared to 41.84% of mostly ordered (chi‐square test, *P* < 2.26E‐146). However, when collagen‐encoding proteins are excluded from this analysis, there is no significant difference in the occurrence of phase symmetry: 42.08 and 41.81% of exons are symmetric for mostly disordered and mostly ordered exons respectively (*P* > 0.133).

**Table 1:** The Percentage of Exons that are Symmetric, for Mostly Disordered and Mostly Ordered Exons and Proteins

	Exon disorder				Protein disorder		
	<30%	>70%	*P* value		<30%	>70%	*P* value
All proteins	41.84%	46.27%	2.26E‐146		41.97%	53.03%	∼=0
Excluding collagenproteins	41.81%	42.08%	0.134		41.92%	44.41%	7.12E‐16

The percentage of exons that are symmetric (i.e., have the same start and end phase). Results are shown for all proteins in the dataset and with collagen proteins removed. Data is shown for mostly ordered and disordered exons on the left, as well as mostly ordered and disordered proteins on the right. *P* values calculated using a Chi‐square test.

If disorder levels are considered for the entire protein, rather than for individual exons, differences in phase symmetry are larger. Of exons found in mostly disordered proteins, 53.03% are symmetric, compared with 41.97% of exons in mostly ordered proteins (*P* = ∼0), which is comparable to previous work.^14^ Excluding collagen‐encoding proteins from this analysis reduces this difference (44.41 and 41.88% for mostly disordered and mostly ordered respectively), though it does remain statistically significant, suggesting there is a modest preference for symmetric exons within proteins that are mostly disordered.

**Figure 3:**
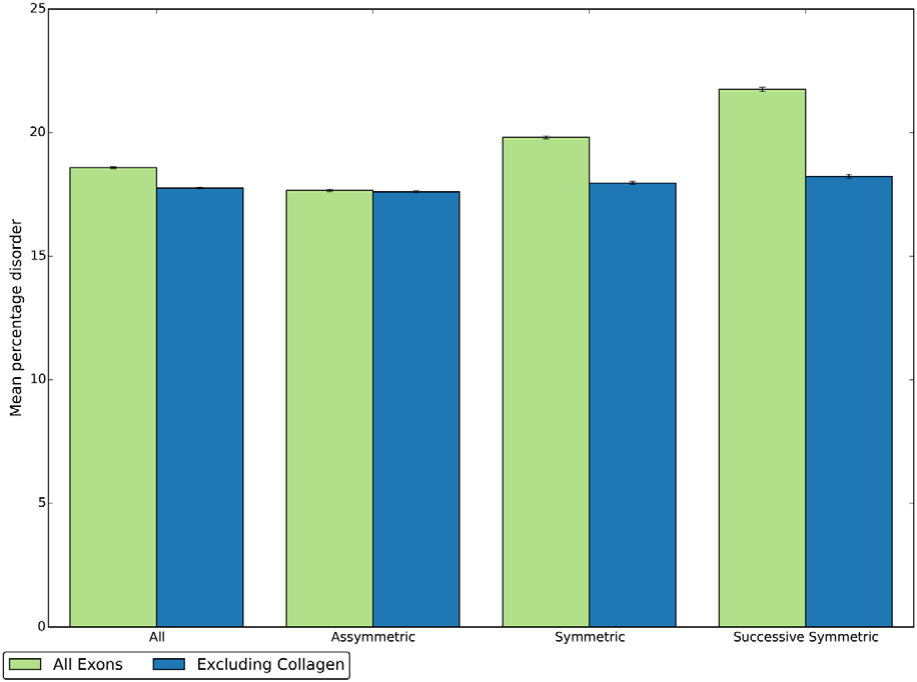
Comparison of mean percentage disorder for different types of exons. Results shown for all exons in the human genome (green) and for all exons excluding those that encode proteins containing at least one Pfam collagen domain (blue). Symmetric exons have the same start and end phase; asymmetric exons do not. Successive symmetric exons are symmetric exons adjacent to exons of the same phase (at least 3 in a row).

This relationship can also be examined by comparing the percentage of residues predicted disordered in symmetric and asymmetric exons.^14^ Figure 3 shows the mean percentage disorder for all, asymmetric, symmetric and successive symmetric exons—the latter is defined as at least three adjacent symmetric exons (which have to have the same phase). There is a significant increase in mean disorder between asymmetric and symmetric exons and again between symmetric and successive symmetric exons (*P* = ∼0 in both cases). If collagen‐encoding proteins are excluded, the mean percentage disorder is highly similar in each type of exon, though the difference between asymmetric and symmetric remains statistically significant. However, since most exons are either fully ordered or fully disordered (Supporting Information Fig. S3), we suggest the chi‐squared analysis shown in the previous paragraph is more appropriate.

### Alternatively spliced exons are enriched for protein disorder, regardless of collagen proteins

**Figure 4:**
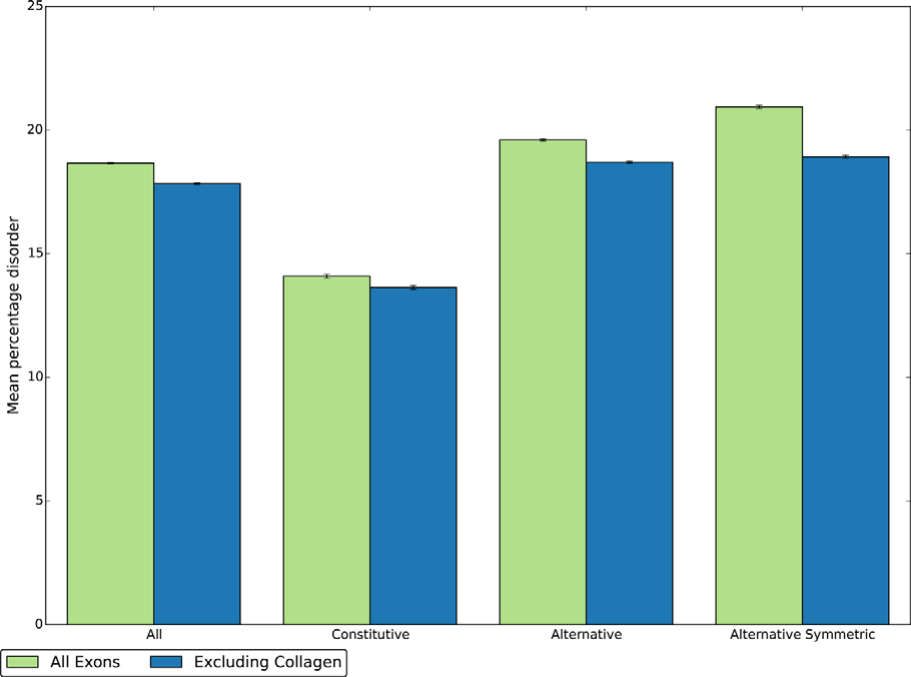
Comparison of mean percentage disorder for different types of exons. Results shown for all exons in the human genome (green) and for all exons excluding those that encode proteins containing at least one Pfam collagen domain (blue). Constitutive exons are exons contributing the same coding sequence from the same genetic locus to all transcripts for a given gene; alternative exons do not.

The enrichment of protein disorder in alternatively spliced exons has been widely reported.^9^, ^10^ Because exons in collagen‐encoding proteins are commonly alternatively spliced, we felt it important to check that this result was not also an artefact of the collagen helix. We find that this result holds true when collagen‐encoding proteins are removed from the dataset. Figure 4 shows the mean percentage of disordered residues for all, constitutive, alternative and alternative symmetric exons. Previous work found exons that are symmetric as well as alternatively spliced are further enriched for protein disorder^14^; however we now find that after excluding collagen‐encoding proteins, there is no significant difference (*P* > 0.04).

### The collagen helix significantly alters the amino acid composition of proteins predicted to be disordered

Although the main focus of this work has been the effect that collagenous proteins have on the properties of exons predicted to encode disordered protein, the promiscuous and compositionally biased nature of the collagen helix means other areas of research may also be impacted.

**Figure 5:**
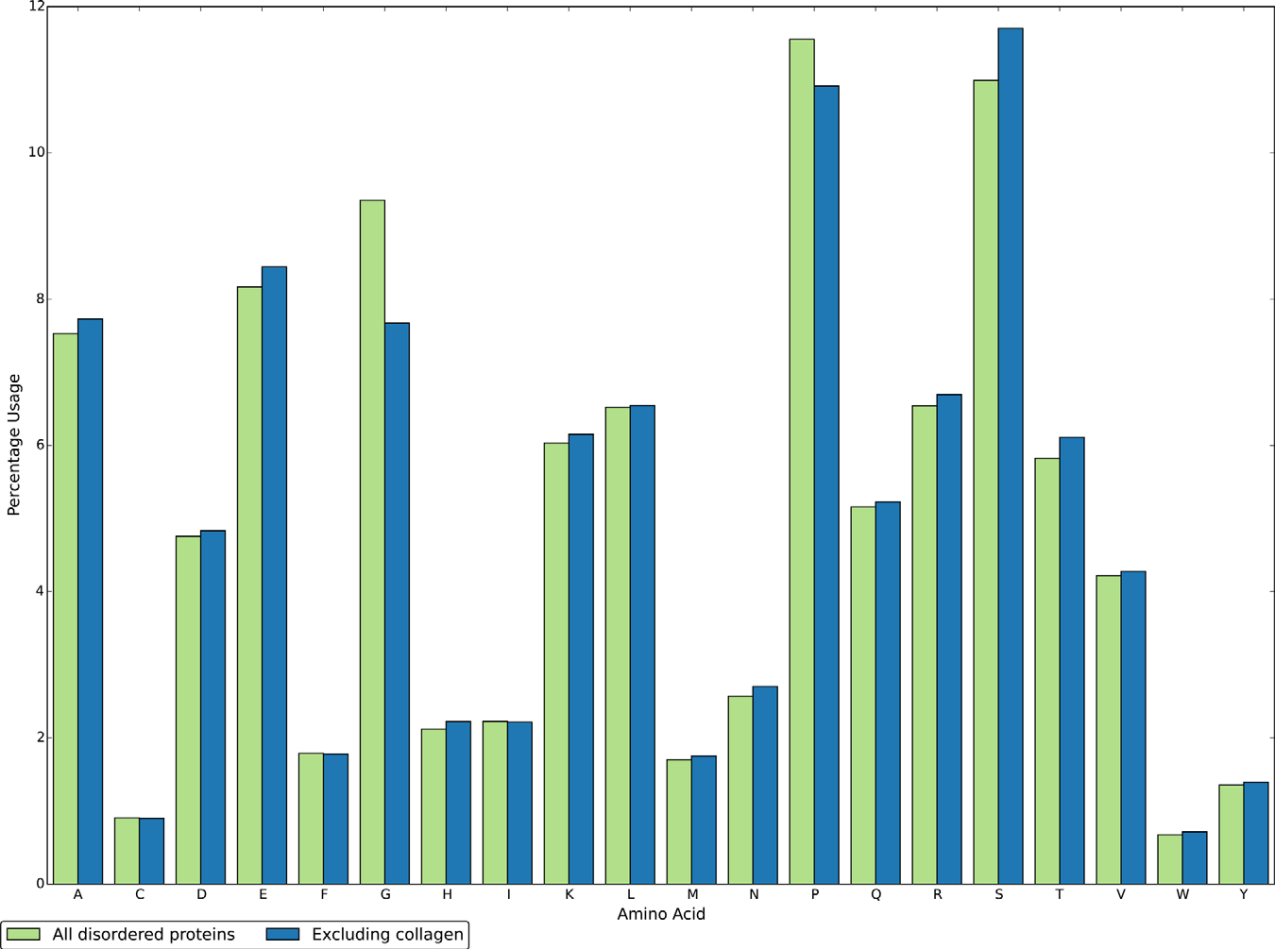
The amino acid composition of all proteins predicted as mostly disordered (green) compared with the composition of mostly disordered proteins excluding those that encode the collagen helix (blue).

To this end, we examined the amino acid composition of proteins that are predicted mostly disordered. Figure 5 shows the percentage occurrence of each amino acid for mostly disordered proteins with and without collagen‐encoding proteins. The occurrence of most amino acids is significantly altered by the inclusion of the collagen proteins (*P* value < 0.0001, calculated empirically from 5000 samples of the distribution), with only cysteine (C), phenylalanine (F), isoleucine (I) and leucine (L) displaying a non‐significant change in usage. In particular, the usage of glycine (G) and proline (P) is strongly overestimated, with serine (S) strongly underestimated.

## Discussion

This work highlights three reasons that the analysis of protein disorder makes more sense in the light of collagen. First, the collagen helix is predicted as disordered despite fibrous protein structure not typically being considered as such. Second, the evolution of collagen through exon duplication skews statistics calculated on disorder‐encoding exons. Third, the repetitive and promiscuous nature of the collagen helix significantly impacts the amino acid composition of proteins predicted as disordered.

The distribution of the lengths of exons that encode disordered protein regions was previously shown to contain a number of peaks (Fig. 3 of Ref. 14), a finding we have replicated here (Fig. 2). We have now identified that many exons of these lengths encode sequences containing the collagen triple helix, and that removing them from the dataset removes these peaks in the distribution. In the same way we also determined that the increased occurrence of symmetry in exons that encode disordered regions can be attributed to the collagen helix, by repeating the analysis of Schad *et al*.^14^ However, we note that the original ideas within this previous work—that phase symmetry has aided modular expansion though tandem duplication of exons—do apply to collagens and other proteins, with no particular preference to disordered proteins as a whole.

Increased levels of disorder in alternatively spliced exons have been widely reported.^9^, ^10^ Our investigation into this observation found that after excluding collagen‐containing proteins, a significant enrichment for protein disorder remains and the observation stands. Nonetheless, we see that the promiscuous and highly repetitive nature of the collagen helix does have some impact unrelated to splicing. We show that the amino acid composition of residues predicted to be disordered is significantly altered by collagen proteins. In particular glycine and proline are over‐represented, as may be expected.

In conclusion, a single structural repeat has the potential to impact a number of analyses to the extent that, in the case of symmetric exons, incorrect conclusions may be drawn. The collagen helix is an extended fibrous protein, a type of structure not typically considered disordered, making this a particular problem. A recent review on the classification of intrinsically disordered proteins shows that there are many faces to protein disorder.^11^ We believe this work further highlights the need for a clearer understanding of what should be considered as intrinsic disorder. In addition, tools for predicting protein disorder could be modified to avoid the misprediction of collagen sequences as disordered. Crucially, the overriding message from this work is that researchers considering the general properties of protein disorder must ensure that proteins that contain the collagen helix are not affecting their results.

## Materials and Methods

Protein sequences and genomic loci of exons from all transcripts in the human genome were obtained from Ensembl (version 63). Exons were then mapped to protein regions using translation start coordinates and exon loci. Any exon not contributing to the coding sequence, that is entirely within the 5′ or 3′ untranslated region, was discarded. After this filtering, the dataset contained 748,665 exons encoding 90,712 protein sequences.

Using D^2^P^2^, regions of proteins predicted as disordered were determined using a consensus prediction: a residue is considered disordered if at least 75% of the nine predictors agree. Using these predictions and the mapping of exons to protein regions, exons were then defined as mostly disordered if at least 70% of the residues they encode were predicted disordered, or mostly ordered if <30% of the residues were predicted disordered. Whole proteins were similarly classified as mostly disordered or mostly ordered using the same thresholds. These thresholds were chosen in line with previous literature.^14^

In addition, end phases for each exon were determined by counting the number of coding nucleotides prior to the end of the exon after the last complete codon. The start phase of an exon is given by the end phase of the previous; the first coding exon is given a start phase of 0. Symmetric exons are those with the same start and end phase. Exons were further identified as successive symmetric if at least three symmetric exons that are adjacent share the same phase.

The collagen Pfam (PF01391^26^) domain was searched against all human proteins using the hmmsearch program from the HMMER package, version 3.0.^27^ Proteins with at least one significant assignment to this domain (*E*‐value <0.0001) were considered collagen‐containing. All exons from each collagen protein were excluded when creating a collagen‐free set of exons.

For each analysis, all transcripts from each gene were used, rather than just the longest transcript. As well as maximizing the dataset, we chose this approach because even where exons are shared between transcripts, this does not imply that the protein level annotation, including intrinsic disorder, will necessarily be identical. In addition, the number of transcripts in collagen genes is not significantly different from other genes (4.9 and 4.2 transcripts per gene respectively, students *t* test *P* > 0.06). Nonetheless, we repeated key analyses using a dataset containing only exons in the longest transcript and found comparable results (see Supporting Information Figs. S4–S6). However, for comparisons between alternative and constitutive exons, only genes with at least two protein products were considered, in line with previous work.^14^ Exons were identified as constitutive if all transcripts from a gene contained an exon with the same genomic locus encoding the same coding sequence in the same phase.

We determined the amino acid composition of mostly disordered proteins and then calculated the difference in this composition when the 155 mostly disordered proteins containing the collagen helix are removed. To assess the significance of these changes, we sampled the distribution by randomly selecting 155 protein sequences to remove and calculating the change in amino acid composition. This was repeated 5000 times and a *P* value calculated using the *Z* score.

Student's *t* tests were used to determine if differences in mean values were significant. Chi‐square tests were used to calculate the significance of differences in the number of exons that are mostly disordered or mostly ordered. A Kolmogorov–Smirnov test was used to compare distributions of exon lengths. In all cases, a significance value of *P* < 0.0001 was used, after Bonferroni correction where necessary.

## Supporting information

Supporting Information
