# Supplementary material for: Three reasons protein disorder analysis makes more sense in the light of collagen
Source: Protein Sci. 2016 Apr 19;25(5):1030–6. doi: 10.1002/pro.2913 (PMC4838654; doi:10.1002/pro.2913)
Supplement: Supplementary file 1 — Supporting Information [file PRO-25-1030-s001.docx]

# Supplementary Material


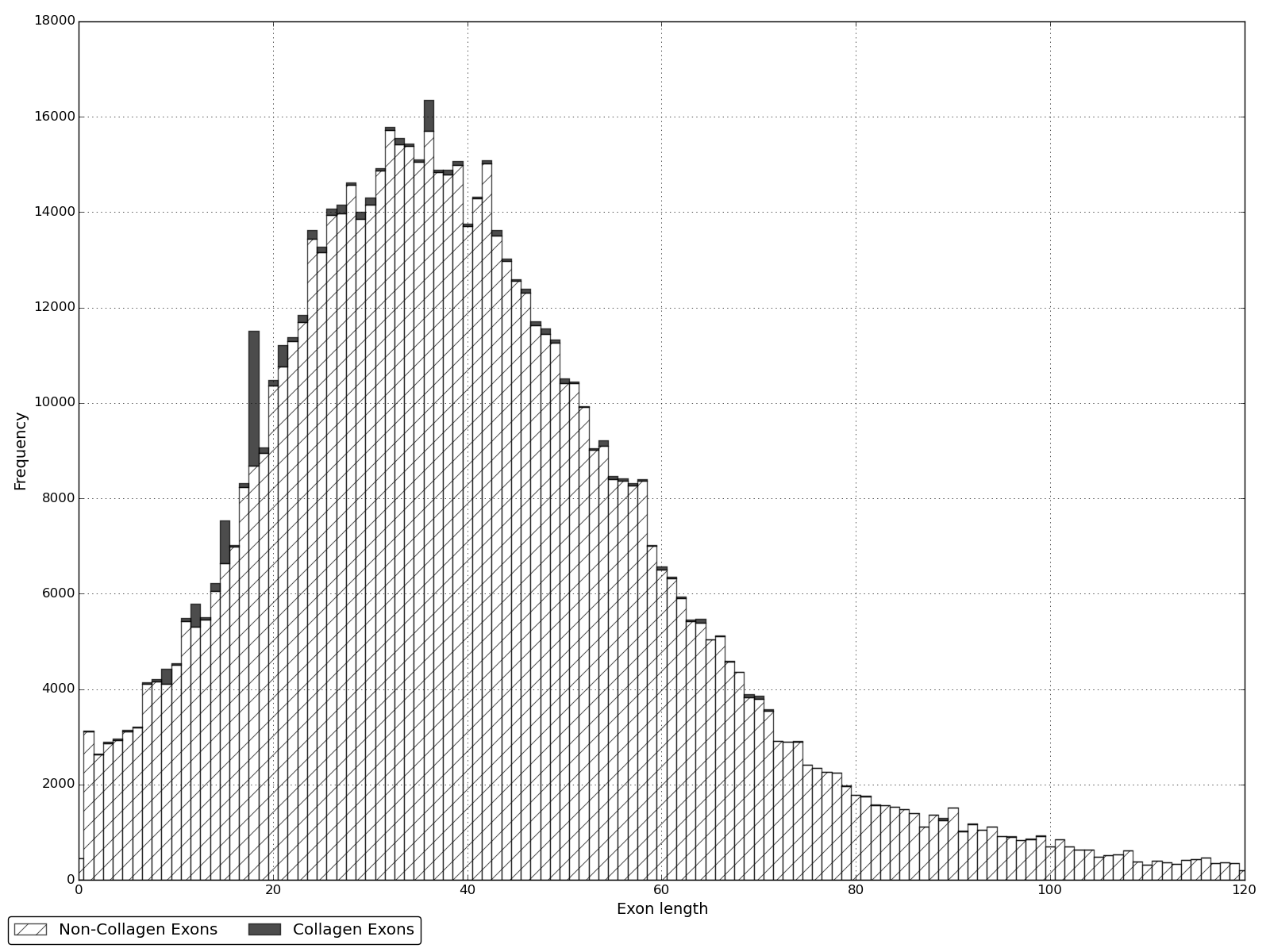


Figure S1: The length distribution of all human exons. Exons that are part of a collagen-containing protein are shown in black; exons not in collagen-containing proteins are show in white (hatched).


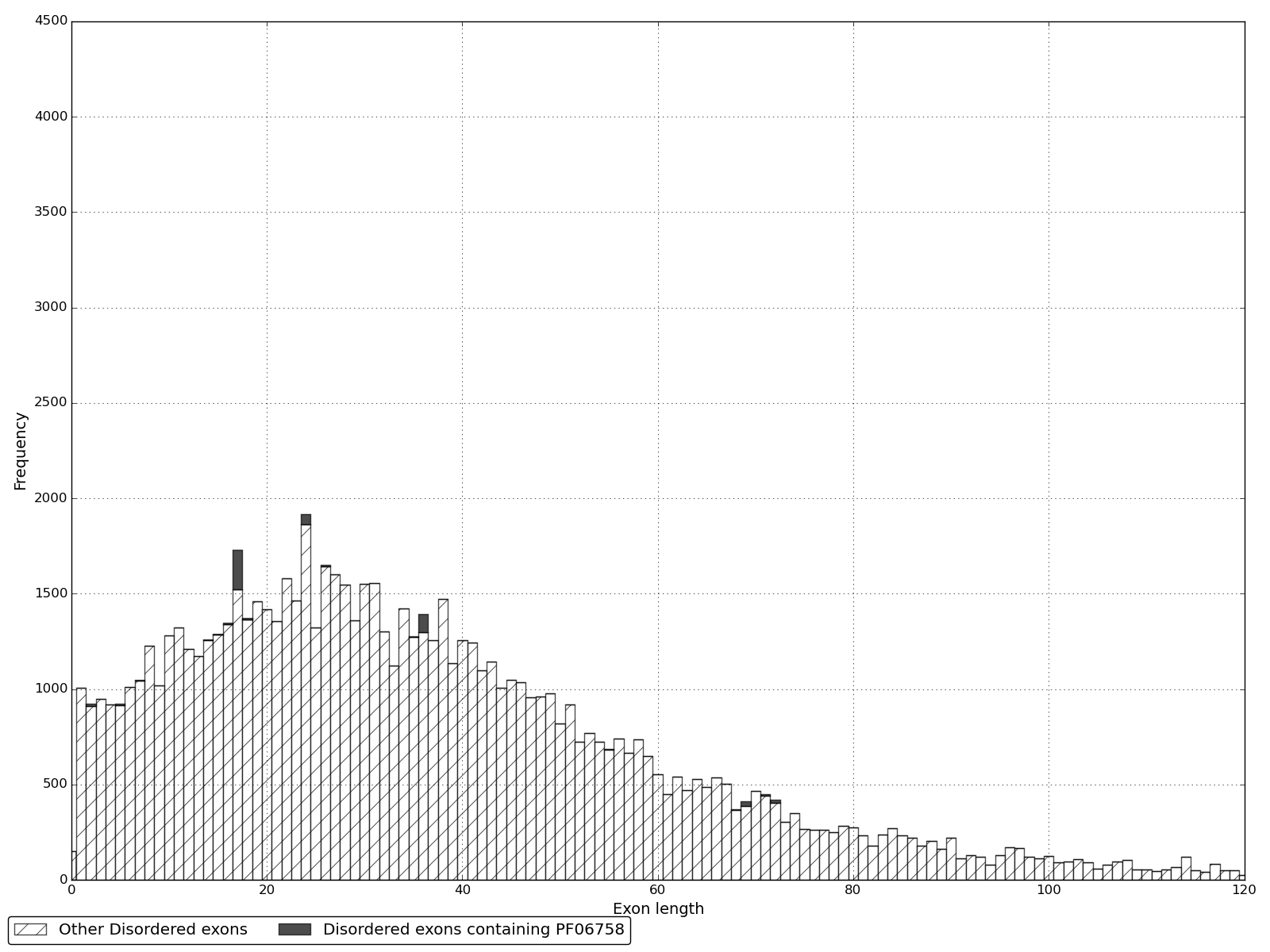


Figure S2: The length distribution of human exons, excluding those encoding collagen-containing proteins. Exons encoding proteins that contain the Pfam domain PF06758 are shown in black, other exons are shown in white (hatched).


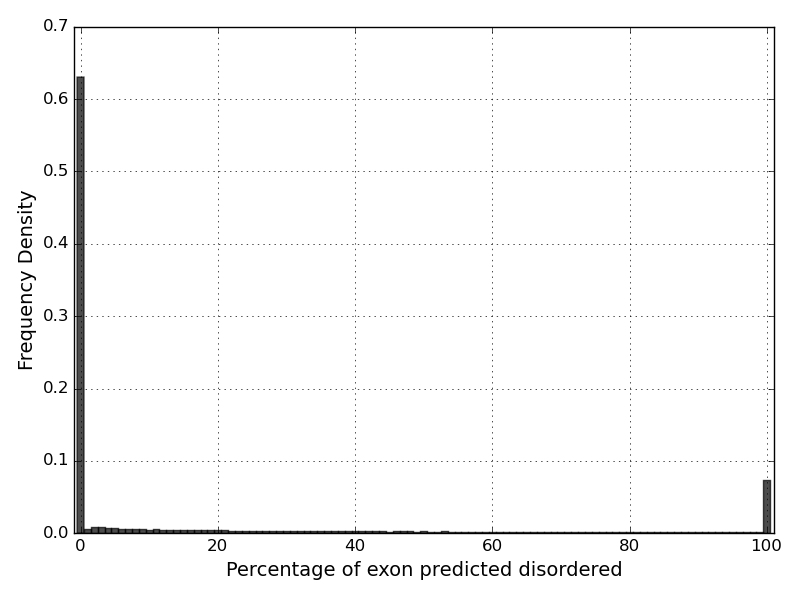


Figure S3: Distribution of percentage of exon predicted as disordered for all exons in the human genome.


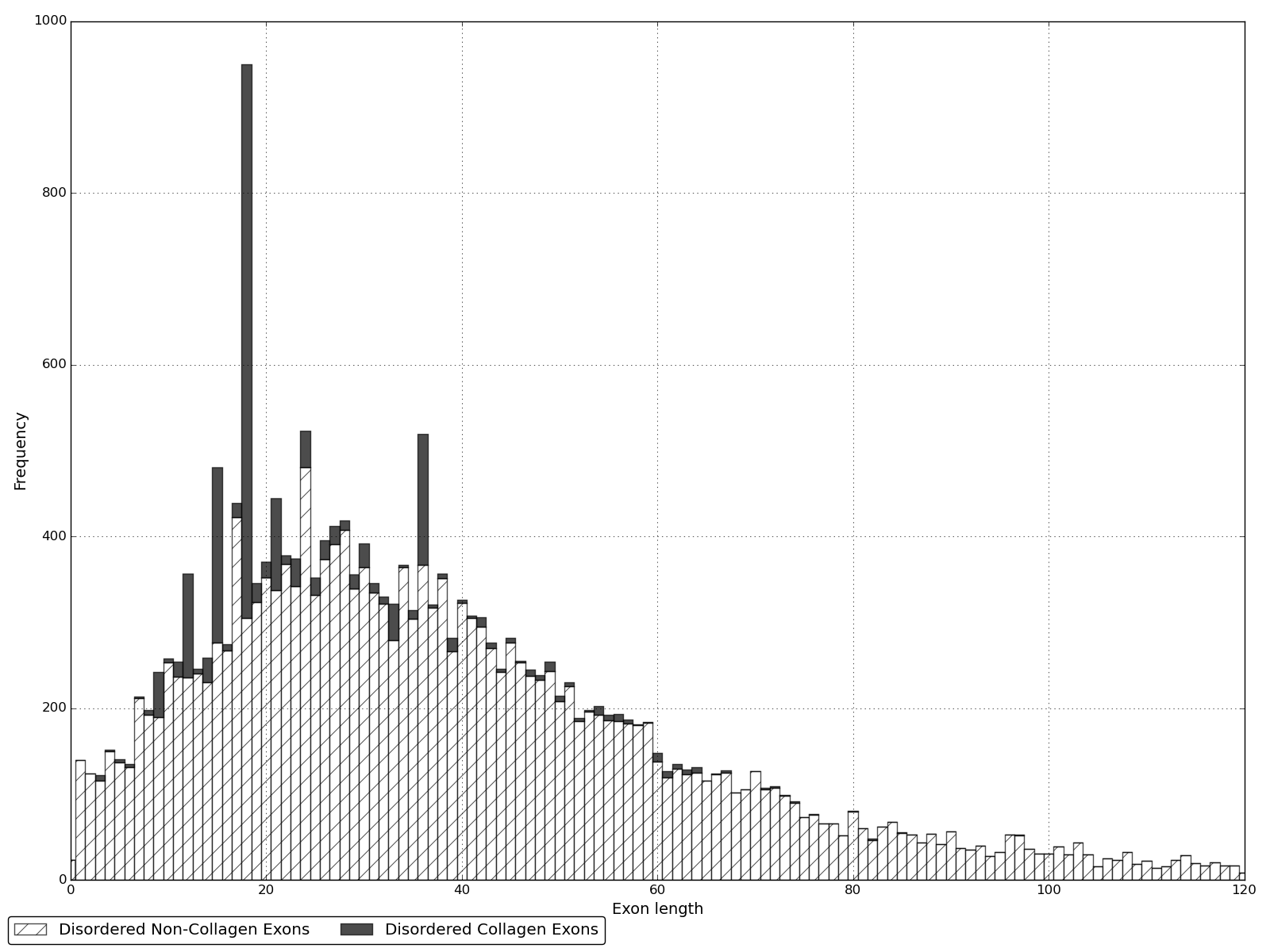


Figure S4: A replication of the analysis presented in Figure 2, only displaying exons that encode the longest transcript in each gene.


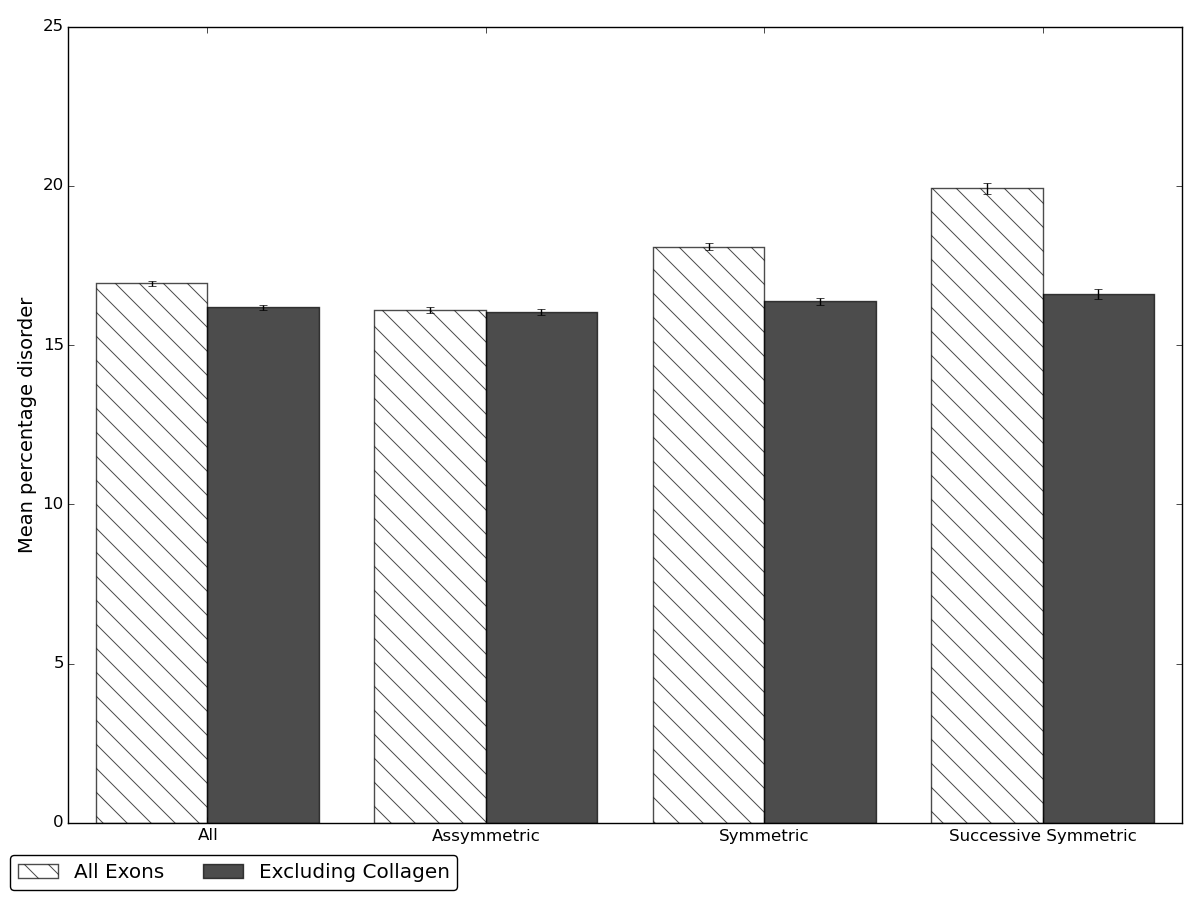


Figure S5: A replication of the analysis presented in Figure 3, only displaying exons that encode the longest transcript in each gene


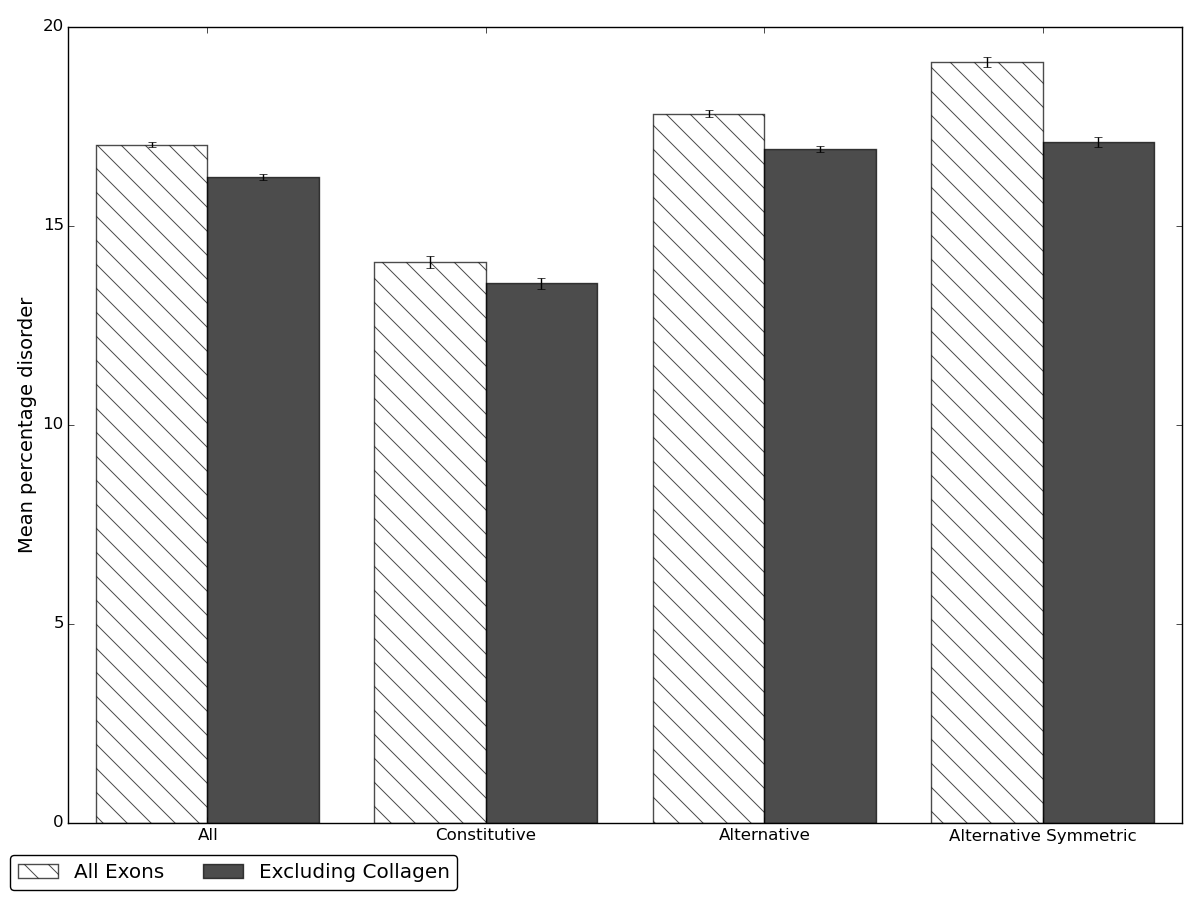


Figure S6: A replication of the analysis presented in Figure 4, only displaying exons that encode the longest transcript in each gene
